# A feasibility study on using soft insoles for estimating 3D ground reaction forces with incorporated 3D-printed foam-like sensors

**DOI:** 10.1017/wtc.2024.23

**Published:** 2025-01-23

**Authors:** Nick Willemstein, Saivimal Sridar, Herman van der Kooij, Ali Sadeghi

**Affiliations:** Department of Biomechanical Engineering, University of Twente, Enschede, The Netherlands.

**Keywords:** soft sensors, soft wearable robotics, exosuits, biomechatronics, mechatronics

## Abstract

Sensorized insoles provide a tool for gait studies and health monitoring during daily life. For users to accept such insoles, they need to be comfortable and lightweight. Previous research has demonstrated that sensorized insoles can estimate ground reaction forces (GRFs). However, these insoles often assemble commercial components restricting design freedom and customization. Within this work, we incorporated four 3D-printed soft foam-like sensors to sensorize an insole. To test the insoles, we had nine participants walk on an instrumented treadmill. The four sensors behaved in line with the expected change in pressure distribution during the gait cycle. A subset of this data was used to identify personalized Hammerstein–Wiener (HW) models to estimate the 3D GRFs while the others were used for validation. In addition, the identified HW models showed the best estimation performance (on average root mean squared (RMS) error 9.3%, 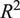 =0.85 and mean absolute error (MAE) 7%) of the vertical, mediolateral, and anteroposterior GRFs, thereby showing that these sensors can estimate the resulting 3D force reasonably well. These results were comparable to or outperformed other works that used commercial force-sensing resistors with machine learning. Four participants participated in three trials over a week, which showed a decrease in estimation performance over time but stayed on average 11.35% RMS and 8.6% MAE after a week with the performance seeming consistent between days two and seven. These results show promise for using 3D-printed soft piezoresistive foam-like sensors with system identification regarding the viability for applications that require softness, lightweight, and customization such as wearable (force) sensors.

## 1. Introduction

With the current boom in the use of advanced health monitoring technologies for both medical and personal use, wearables have become a large part of day-to-day lives [1]. The use of smartwatches and heart rate monitors for sports, and recreational activities, as well as monitoring of health conditions, is steadily on the rise to maintain optimal physical performance [2]. Biological markers such as cardiac rhythm, blood pressure, and respiratory variables have been investigated using smartphones and smartwatches [3, 4], which could aid in the detection of, for instance, atrial fibrillation [5]. Although useful, these technologies are limited to the estimation of bio variables by the type of sensors available for use [6].

In the field of kinesiology, the estimation of moments about the joints in the human body has been vastly studied for understanding human biomechanics as well as applying them to sports science and wearable robotics. For the estimation of biological joint torques, the use of motion capture systems in conjunction with force plates has been widely utilized [7]. This setup is highly accurate but cannot be utilized in outdoor scenarios. Therefore, wearable sensors offer several benefits over commercially available smart devices such as phones and watches.

Typically, inertial measurement units have been utilized to estimate the kinematics of the human body [8]. In such setups, the inertial data is fitted to models with person-specific limb mass and center of mass locations. Such models make assumptions that might not represent certain populations, such as the elderly or obese [8, 9], thereby requiring complex equipment such as 3D scanners to derive a proper model [9].

Therefore, several groups have worked on designing insole sensors for measuring ground reaction forces (GRFs) via the use of several types of sensors. Commercially available load cells have been utilized for integrating sensing into insoles [10] but have the downside of being bulky. A solution to overcome this downside is to use soft/flexible sensors. For instance, by using air pressure sensors embedded in the shoe [11], capacitive sensors embedded in textiles [12] and piezoresistive sensors [13]. However, these insoles were often limited to the measurement of vertical GRF and not the mediolateral and anteroposterior GRFs. Typically, these are realized using manufacturing processes such as molding [14] and manually coiling a tube [15]. More recently, researchers have been investigating 3D printing, which can realize high geometric complexity without requiring complicated processing steps.

Due to advances in additive manufacturing, it is now possible to 3D print compliant materials such as thermoplastic elastomers [16] and use conductive polymers [17] for sensing applications. Moreover, 3D printing has the potential to create patient-specific solutions and reduce foot-related musculoskeletal ailments [18]. Furthermore, printing with a sensorized material could allow for personalized insoles that can estimate the GRFs.

Some researchers have already investigated 3D-printed insoles with integrated sensing [19, 20, 21, 22]. One approach is to embed 3D-printed electrodes separated by a dielectric to act as a capacitive sensor [20, 23, 24]. Another method is to 3D print the insole and embed optical fibers [21] or force-sensing resistors (FSRs) [19]. The latter approach uses piezoresistive sensing, which requires simple read-out circuitry. This piezoresistive sensing was also shown to be possible in the 3D-printed soft insole [22].

A challenge when using piezoresistive sensors is their inherent nonlinearities and hysteresis making the relationship between resistance change and the stress not straightforward. To convert the resistance change to stress, researchers have investigated machine learning [25, 26, 27] for insoles with embedded commercial FSRs. This approach was shown to work for estimating the 3D GRFs [26, 27]. Another approach is to use system identification, which can give models with (relatively) lower computational complexity. System identification of Hammerstein–Wiener (HW) models has been investigated for not only commercial FSRs [28] but also strain sensing of 3D-printed piezoresistive sensors [29]. However, 3D-printed foam-like (that is porous) piezoresistive sensors for 3D GRF estimation have not, to the authors’ knowledge, been investigated.

The combination of sensing and foam-like behavior makes them an interesting candidate for sensorized insoles. The foam-like structure makes such sensors programmable in terms of stiffness [30], which can be used to fabricate patient-specific insoles. For example, such insoles could be used for diabetic patients to reduce foot ulcers [31], which can be augmented with foam-like sensors to provide more information.

Thus, researchers have shown that insoles incorporating FSRs can estimate GRFs [26, 25, 27]. In contrast, 3D-printed insoles can sense and be customized [20, 21, 22, 23, 24] but have not been used to estimate the 3D GRFs. In this work, we investigated the feasibility of using 3D-printed sensorized insoles to estimate 3D GRFs. Specifically, we investigate how to convert the raw output of 3D-printed foam-like sensors to 3D GRFs and compare to FSR-based insoles. Furthermore, we combine this with the softness of foam-like sensors for comfort and mechanical programmability [30], thereby moving towards 3D-printed sensorized insoles that can estimate 3D GRFs. In addition, the sensors’ softness allows for integration into an insole while not feeling like an external element. This study can act as a stepping stone toward 3D-printed insoles that can be personalized and sensorized.

Compared to other sensorized insoles in literature, the presented insoles leverage additive manufacturing of soft thermoplastic elastomers to make foam-like sensors using our InFoam Method [30]. An advantage of the InFoam method is that it can set the stiffness by adjusting the porosity magnitude [30]. In addition, it can make the printed structure softer than the base material, which should improve comfort and allow the realization of patient-specific solutions [31]). Lastly, it does not require any postprocessing or chemicals to sensorize the structure, making fabrication straightforward. In this work, we will show that combining the InFoam method with system identification allows for 3D GRF estimation, thereby providing a manufacturing and calibration method for soft sensorized insoles that can estimate the GRFs. Furthermore, the presented approach of combining 3D-printed foam-like structures and system identification could be useful for soft wearable force sensors.

This article is organized as follows. Section 2 details the design of the soft sensorized insole, the force estimation method, its fabrication method, and the test procedure used to evaluate the insole. Sections 3 and 4 present the results and discussion, respectively. These sections discuss the results in terms of sensor behavior during a cyclic load, the gait cycle, and the estimation of the GRFs (vertical, mediolateral, and anteroposterior) for personalized models for a single and multiple day(s). In addition, a subsection in the discussion is dedicated to comparing our insole to other insoles in literature. Lastly, the conclusions from this work and future work are detailed in Section 5.

## 2. Methods

### 2.1. Soft sensorized insole design and fabrication

**Figure 1.**
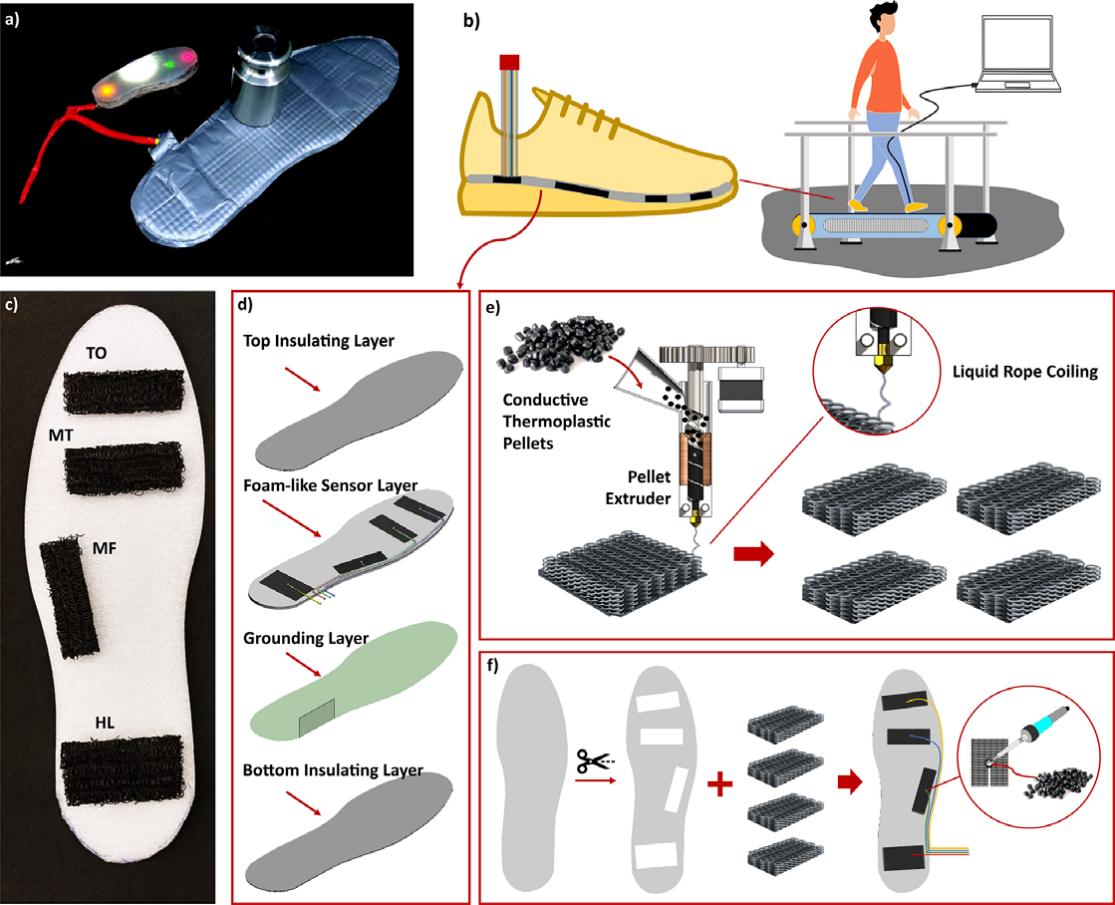
(a) The sensorized insole while measuring the application of a force due to the foam-like sensor deforming reducing the resistance and increasing the LED’s brightness, (b) we evaluated the insole of these insoles on an instrumented treadmill, (c) the foam-like sensors were located on the toe (TO), metatarsal (MT), midfoot (MF), and heel (HL). (d) The sensorized insoles are composed of four layers, (e, f) the fabrication approach for realizing the insole, which includes (e) combining 3D printing and liquid rope coiling to create foam-like structures and (f) subsequent assembly and soldering steps.

Realizing a sensorized insole requires the integration of sensing into a flexible/soft foot-shaped structure. The softness of foam-like structures can be very promising for such an application. Foam-like structures have been investigated in literature and were shown to be very promising due to their large change in resistance when a force/strain is applied [29]. This piezoresistive effect is also used in this work. The significant change in resistance can also be seen in Figure 1(a). Due to the large change (>75% possible, as will be shown later) in resistance, the LED can go from dim to very bright, thereby realizing a sensitive yet soft sensor.

In this work, foam-like sensors are used for sensorized insoles to measure GRFs. Specifically, we focus on using the insoles on an instrumented treadmill (Figure 1(b)). To reconstruct the GRFs, the sensors need to capture enough data during walking. To this end, the sensors were distributed over the entire length of the foot. Specifically, the sensors were added at the heel (HL), midfoot (MF), metatarsal (MT), and toes (TO) (Figure 1(c)). These locations should allow for the reconstruction of aspects of the gait cycle such as heel strike and toe-off. Furthermore, these locations experience most of the pressure on the foot during walking thereby aiding in GRF estimation.

The complete insole also required the addition of insulation to separate the user and conductive components. To this end, an insole was designed consisting of four individual layers (Figure 1(d)): a soft foam layer that integrates the sensor, two insulating (top and bottom) layers, and a layer with grounding electrodes. The purpose of the insulating layers was not only to prevent the user from creating an additional conductive path but also to package the entire structure.

The sensing of the insole was accomplished by the grounding and sensor layer. The sensor layer consisted of four piezoresistive foam-like (porous) sensors (Figure 1(c)). These piezoresistive sensors [29] change their resistance when subjected to a load. The resistance of the sensors was measured using a voltage divider with the foam-like sensor inside the shoe and a second resistor external to the shoe. To complete the voltage dividers, a common grounding layer was added inside the insole. A small strip was added at the edge of the common ground layer to provide a connection point for the wiring outside of the user’s shoe.

These sensors need to be soft and lightweight to be acceptable for wearable sensing applications. To adhere to this requirement, the sensor was made from a soft foam-like (porous) structure. The foam-like nature makes it both soft and lightweight. These porous sensors can be realized using 3D printing through our InFoam method [30]. This method exploits the liquid rope coiling effect, which is the coiling effect also seen when dropping viscous liquid such as honey from a certain height. The empty space of the coils creates pores leading to a structure with programmable porosity. By printing this foam-like (porous) structure out of a conductive thermoplastic elastomer, the structure can act as a piezoresistive sensor [29].

The insole was fabricated using the approach shown in Figure 1(e) and (f). The first step was to print the porous sensors, which were printed using our InFoam method [30]. Within this work, a modified Ender 5 Plus (Shenzhen Creality 3D Technology Co., Ltd., China) with a custom screw extruder was used. The screw extruder used TC7OEX-BLCK pellets (Kraiburg TPE, Germany) that have a Shore Hardness of 70A and volume resistivity of 10 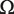 cm. These were printed at 195 °C with a 0.6 mm nozzle. Using the conductive thermoplastic elastomer (cTPE) pellets, the piezoresistive sensors were printed with sizes of 60 mm by [20, 25, 30] mm porous blocks for the toe, metatarsal, midfoot, and heel sensors, respectively. The foam-like sensor had a height of 8 mm and a porosity of 73%, which was experimentally determined to not be noticeable by the participant when integrated into the insole.

In the second step (Figure 1 (f)), a sheet of polyethylene foam (thickness of 5 mm) was cut to shoe size 43 (EU). Four pockets of appropriate size (see above) were cut at the toe, metatarsal, midfoot, and heel for embedding the sensors. Subsequently, the 3D-printed sensors were placed in each pocket. An electrode (a copper wire) was attached to every sensor. These wires were soldered to the sensor by using cTPE pellets. These wires were guided through the insole’s edge by using tape to have a single point of entry at the lateral malleolus.

Afterwards, the common grounding layer was added. This common grounding layer consisted of a woven sheet of conductive textile (Adafruit, USA) that was connected to the Arduino. This piece of textile had a piece that stuck out near the lateral malleolus. Lastly, to finalize the insole, an insulating layer was added on top and the bottom of the insole by wrapping it in insulating tape.

### 2.2. Force estimation by identified models

The GRFs are estimated by combining the resistance change of the four piezoresistive foam-like sensors. These sensors change resistance when subjected to a force by the collapse of the porous structure reducing the resistance. Within this work, the change in resistance 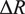 (%) is used for force estimation. The change in resistance at timestep 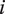 is defined as (with 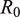 the resistance when the user’s feet are in the air (that is zero-load))
(2.1)

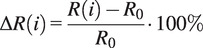

**Figure 2.**
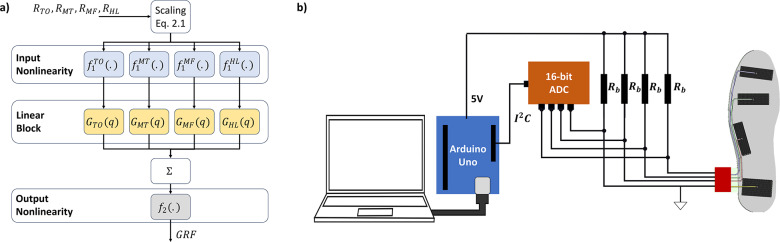
(a) The Hammerstein-Wiener model’s pipeline that converts the resistance change of the sensor to a ground reaction force (GRF), and (b) the measurement setup of the right sensorized insole using an Arduino and a four-channel 16-bit analog-to-digital converter (ADC) connected through I 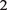 C, for the left insole a second ADC was added and connected in the same way to the same Arduino.

This change in resistance can be exploited to estimate the gait cycle behavior. Unfortunately, the change in resistance of piezoresistive porous sensors is nonlinear and exhibits hysteresis. These nonlinearities need to be compensated for to estimate the GRFs. In literature, people solve this by using a model-based solution. Within this work, HW models are employed as they require a relatively low number of parameters yet can still provide good force estimates, as seen in [28]. The HW model is a class of nonlinear models that uses a block-oriented structure. The HW model consists of three blocks as seen in Figure 2(a).

Following a single input, the HW model includes a linear ( 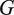 ) and two nonlinear functions ( 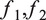 ). Firstly, the linear transfer function 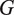 is defined as (at time step 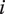 )
(2.2)

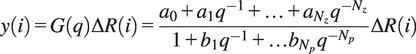

This transfer function uses the shift operator 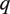 defined as 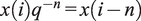 (i.e. 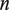 samples before the current time step) with 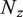 zeros and 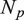 poles.

Besides the linear transfer function, HW models incorporate the nonlinear functions 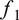 and 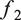 . These functions can be any nonlinear function such as a polynomial or a neural network. Within this work, piecewise linear functions were used as these gave good results for strain [29] and force estimation [28]. The number of breakpoints was determined experimentally by trying different combinations in the range of five to ten. After initial manual model estimation, the amount was set to ten breakpoints.

Combining multiple inputs using 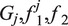 , we can define the output force as
(2.3)

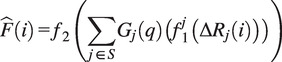

where the variable 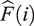 represents the estimated GRF at timestep 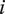 while 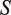 is the set of sensors ( 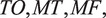 and 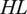 ). Note that the nonlinear input function 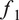 consists of four nonlinear functions (one for each sensor). The HW models are identified using MATLAB’s System Identification Toolbox (The MathWorks, USA).

Before model estimation, the data were detrended using MATLAB’s detrend-function to make system identification easier [32]. Specifically, we removed the mean of the data to make both the resistance changes and GRFs centered around 0. Afterwards, we removed the linear trend for drift using detrend as well. The mean was added back for plotting.

Models were estimated for each GRF separately, which required the estimation of a set of three multi-input–single-output systems per insole. In addition, models were identified for each participant individually for personalized models, which mirrors the approach for a patient-specific insole. To determine the optimal model orders, we employed a brute-force strategy that iterated over a set of pole-zero combinations. To reduce the parameter space, we set the amount of pole-zero combinations equal for all inputs. For the linear models, we explored all the causal pole-zero combinations from one to ten poles. In contrast, the HW models had a slightly limited scope in terms of pole-zero combinations based on initial manual model estimation. This manual exploration led to the restriction of one to eight poles and one to five zeros. Furthermore, only causal pole-zero combinations were considered, which were run twice to reduce the odds of getting an unfavorable initial condition.

After model estimation, the models were evaluated using their root mean squared (RMS) error and mean absolute error (MAE) scaled by the maximum change in body-weight normalized force. These errors were defined as
(2.4)

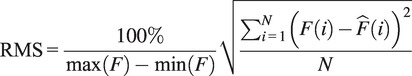



(2.5)

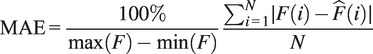

Within this equation, the variables 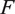 and 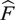 represent the measured and estimated force, respectively, whereas 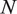 is the number of time steps. In addition, the coefficient of determination 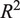 was computed by comparing the measured and estimated GRFs. The best model was selected based on the lowest RMS error. Furthermore, to enable comparison between multiple participants, the GRFs are scaled by the body weight of the participant.

### 2.3. Heel sensor testing

To evaluate the behavior of the insole under repeated loading, we performed a cyclic compression test on the heel sensor. We chose this sensor as it would experience the highest force during walking. To mimic loading during the walking cycle, we used an Instron 3,343 tensile tester (Instron, USA) and applied a 5-mm deformation at 800 mm/min for 10,000 cycles. During this experiment, we recorded the resistance change using the setup of Figure 2(b) while the Instron recorded the force.

### 2.4. Sensorized insole testing

Two sensorized insoles, weighing 31.1 grams each and having EU size 43, were fabricated that could be embedded in a shoe for testing. The wiring of the insole was attached to the subject by using elastic bands with velcro to ensure that the wiring did not influence the walking motion. All participants walked on a dual-belt, force-instrumented treadmill (Bertec Corporation, USA) with integrated force plates (Advanced Mechanical Technology, Inc., USA). The instrumented treadmill was used to record the GRFs of both feet. Walking trials were performed on the treadmill with both increases and decreases in speeds in a trapezoidal pattern. Specifically, the trapezoidal pattern of 2.5, 3, 3.5, 3, and 2.5 km/h was used, which each lasted approximately 50 seconds. This walking trial was performed three times.

To investigate the applicability of our sensorized insole, nine participants were recruited. Before recruitment, the experimental procedure was approved by the Natural Sciences and Engineering Sciences Ethics Committee of the University of Twente (reference: 230464). Nine healthy participants (1 female; age: 29.2 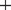 6.5, weight: 80.1 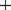 10.1 kg, height: 1.80 m 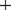 0.04 m, and shoe size 43.2 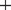 1.2) with no abnormal gait pattern participated in the study after giving written informed consent. Although participants had different shoe sizes, the insole was soft and could fit inside all the shoe sizes (EU 42 to 45) without any issues. Moreover, the sensors should be primarily affected by the intended anatomical location, as the foot length (based on shoe size) varied between 260 and 282.5 mm. At most this foot length change is [−9,+13.5] mm compared to EU43 (269 mm), which is within the length of the smallest sensor (20 mm).

All participants were given the same set of insoles with one dedicated for the left and one for the right shoe. They embedded the insole in their shoes. During a single experiment, the participant performed three walking trials. The first two walking trials were used for identification. Whereas the third trial was a validation dataset for the personalized models. This experiment was performed with all participants. Four of the nine participants did this experiment over multiple days. They came back for a second and third experiment of three trials on the day after and one week after. This multiday experiment was performed to provide insight into whether the model performance stayed consistent over time. During all of the walking trials, the changes in resistance were recorded using the setup shown in Figure 2(b) for the right insole.

The measurement setup (Figure 2(b)) used a voltage divider to measure the resistance of the sensor using a bias resistor 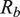 of 560 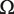 . The output voltage was measured using a 16-bit analog-to-digital converter ADS1115 (Texas Instruments, USA) on a breakout board (Adafruit, USA) that is connected to an Arduino Uno (Arduino AG, Italy). The Arduino streamed data over USB to a laptop for further processing. The measured voltages were then converted to resistance by
(2.6)

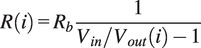

Within this equation, the variables 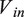 and 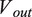 represent the input (5 V from Arduino) and measured voltage, respectively. The resistance changes were then computed by using 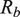 and Eq. 2.1. The changes in resistance were then used as the inputs of the to-be-identified linear and HW models for each participant.

## 3. Results

### 3.1. Cyclic experiment result

**Figure 3.**
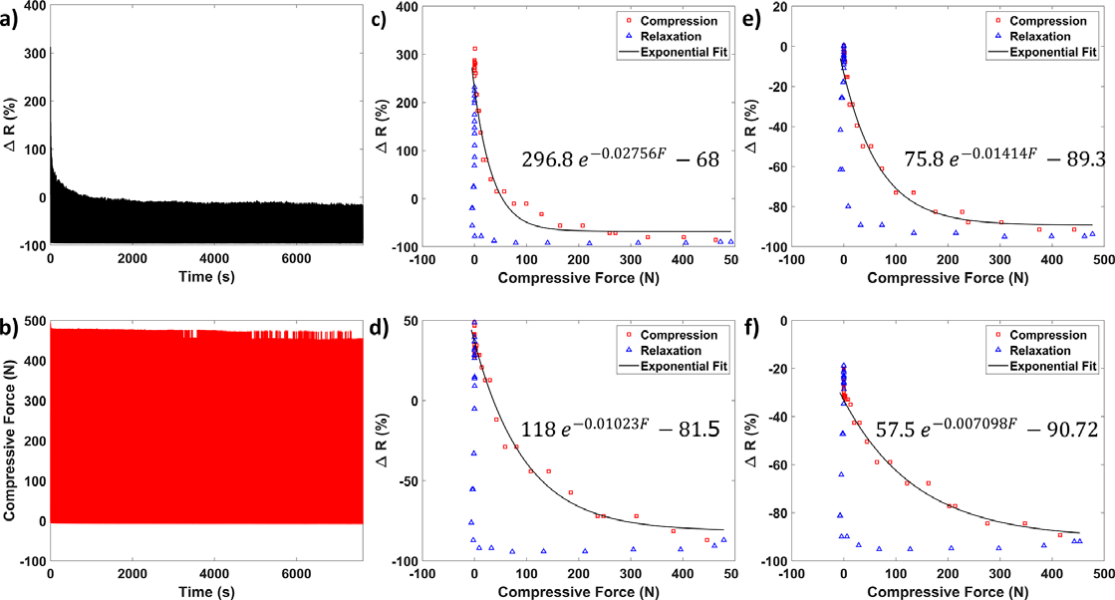
Cyclic experiment results with (a) resistance change and (b) compressive stress over cycles, (c-f) hysteresis plots with exponential fit parameters for cycle (c) 1, (d) 10, (e) 1,000, and (f) 10,000.

The results of the cyclic test (Figure 3(a)) show that the stress decays over the cycles. This viscoelastic relaxation is expected for a thermoplastic material. The stress decays rapidly to a near steady-state value within 30 seconds (around 38 cycles).

The resistance change’s minimum (under maximum load)-value stays around −90%. We see a charging-like behavior as the no-load resistance change (that is the maximum value) drifts until reaching a stable value, which was also observed in our earlier work in a sensorized actuator [29]. The trend implies that the sensor is charging, which could be explained by the individual carbon-black particles inside the TPE acting as a resistor and capacitor in parallel [33]. Over time, the capacitors will charge up leading to a drift. This charging does seem much slower than the stress relaxation around 1,000 and 3,000 seconds for the largest change and near final value (1,300/3,800 cycles).

In addition, the relation between resistance change-stress displays hysteresis (Figures 3(b–d)), which reinforces the need for a model-based solution. The behavior of cycles 1,000 and 10,000 seem very similar but the exponential fit does change a bit between these cycles, although there is less change between them than between cycles 1 and 10. Furthermore, the plots show no indication of sensor failure.

The sensitivity was evaluated by the fitted exponential curve to the hysteresis cycles 1,100,1,000, and 10,000 (Figure 3(b–d) for the compression cycle from force to resistance change. The slope of this curve is the sensitivity of the sensor, which seems higher for lower force and reduces rapidly. An explanation hereof is that a larger change in surface contact happens when the pores buckle at lower forces. Due to the resistance being at least zero Ohms, the maximum resistance change is −100%, which limits the sensitivity at higher forces. In addition, one can observe that the sensitivity (see Supplementary Materials) to low forces decays and stabilizes with cycles 1,000 and 10,000 being similar. In addition, the sensor seems to become less sensitive to low forces over time. However, it is unclear whether it is due to viscoelastic relaxation, charging, or a combination thereof.

The pressure range of the sensor was computed by extrapolating from the curve when −100% resistance change is reached (that is fitting an exponential curve from resistance change to force). This approach gave a value from 600 N (first cycle) to 810 N (last cycle), which is on average 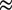 705N. This magnitude shows that the four sensors combined can hold and measure the expected vertical load (>2,810 N load possible). This force magnitude is less than the expected vertical load during heel strike (950 N on average for our participants). However, this analysis does not account for the insole, which would provide additional support, and is much stiffer than the foam-like sensor, which should limit saturation issues.

### 3.2. Sensor behavior during gait cycle

**Figure 4.**
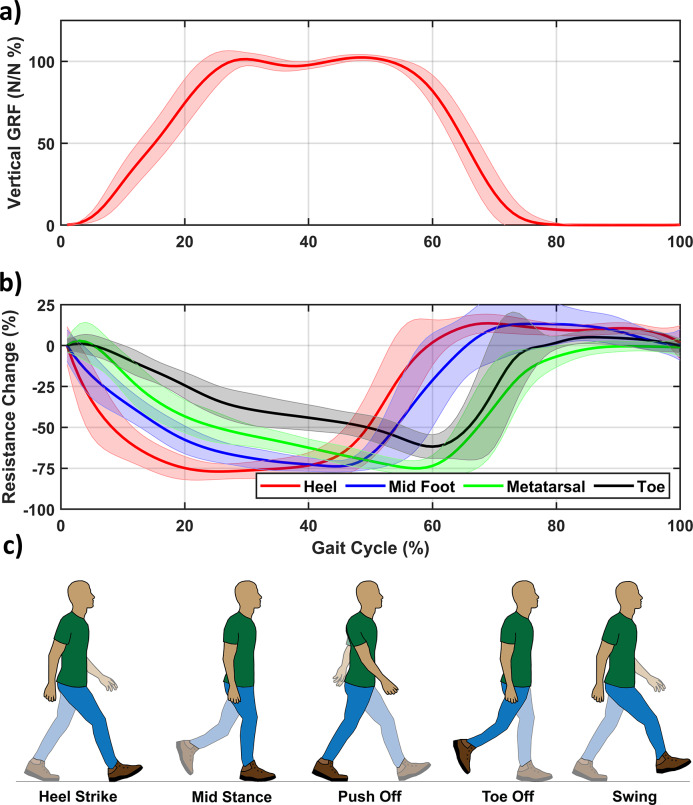
Sensor behavior during the gait cycle of vertical forces (a), resistance change (b), and gait phase (c) for one of the participants.

The vertical GRF versus gait cycle is shown in Figure 4(a,b) in error bar format based on a spline fit. A typical gait cycle can be observed with a clear heel strike ( 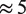 %), mid stance ( 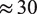 to 50%), and toe-off ( 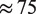 %) [7]. The resistance change curve (Figure 4(b)) has an overall similar shape as the gait cycle but is inverted. The inverted shape is to be expected as the piezoresistive sensors decrease in resistance with increased force.

The sensor behavior over the gait cycle has similarities in their general shape to the vertical GRF. The sensors reach peaks and relax in line with the change in pressure distribution expected over the gait cycle (Figure 4(c)). From heel strike to mid-stance a successive activation from the heel to toe is seen, which indicates a gradual (expected) change in force distribution from the heel to the entire foot during mid-stance. After the mid-stance, the sensors at the midfoot, metatarsal, and toe sensor show an increase indicating that the subject is shifting weight towards his toes (as expected). Moreover, during this phase, all sensors show relaxation in the same order as the heel strike event (that is from heel to toe). Lastly, the shift in force towards the metatarsal/toe should be right before the toe-off, as also seen in our sensors.

### 3.3. Single day trials

**Figure 5.**
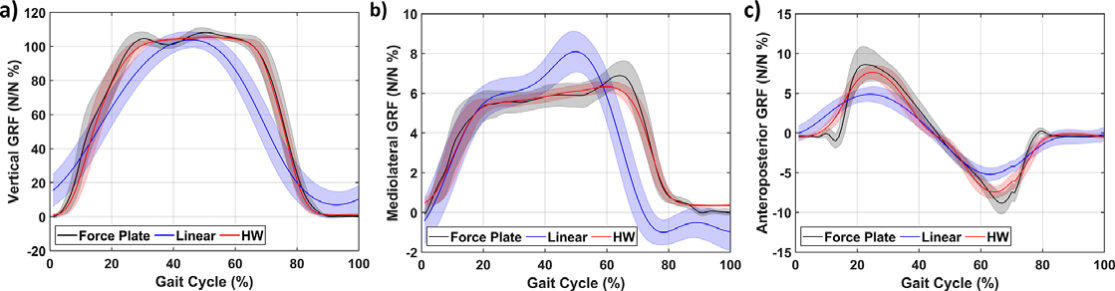
Estimated and measured 3D ground reaction forces based on the segmented gait cycle for one of the participants for the (a) vertical, (b) mediolateral, and (c) anteroposterior forces averaged based on approximately 115 cycles on the day of model identification.

Using the data collected during the first day of trials, a set of HW and linear models were estimated. The estimated GRFs by these models are shown for a segmented gait cycle (averaged over all the gait cycles during the walking trial) in Figure 5(a–c). The vertical, mediolateral, and anteroposterior GRFs are decently approximated by the HW model in terms of magnitude. The linear models seem to have difficulty with the vertical GRF, while the other two seem more reasonable but worse than the HW models. In general, the results for the averaged GRFs presented in Figure 5 show that the insole can reconstruct the GRFs’ shape quite well. However, the estimated values do not show the peaks of the GRFs, which were captured by the force plate data indicating some discrepancies.

The level of accuracy was further explored in terms of RMS, MAE, and the coefficient of determination ( 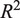 ). The estimation quality was examined in terms of both the segmented gait cycle and the entire time series. These two perspectives were employed as segmentation is useful for clinical purposes whereas time-series estimation is more important for closed-loop control of, for instance, an exoskeleton. Segmentation provides less insight for the latter due to the averaging inherent to segmentation can obfuscate outliers. These outliers can negatively impact closed-loop control. The model selection of both was done separately to investigate the effect of the model order on either application.

**Figure 6.**
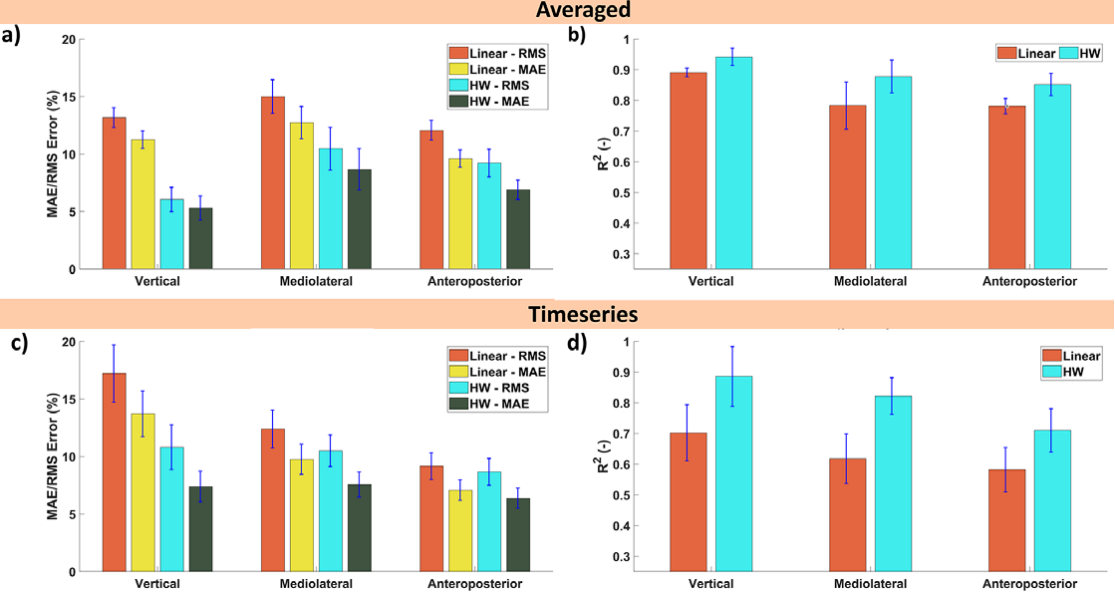
Estimation performance on the same day as model identification with standard error of the ground reaction forces with the RMS error and MAE as a percentage of the force amplitude and the coefficient of determination 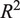 for both the (a,b) segmented gait cycle and (c,d) time-series for the HW and linear models.

Both of these results are shown in Figure 6. For the segmented gait cycle (Figure 6(a) and (b)), it can be observed that the HW model performs well with RMS errors being 6.1, 10.5, and 9.2% for the vertical, mediolateral, and anteroposterior GRFs, respectively. Similarly, the MAE and 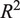 also indicate good estimation performance with on average 7% and 0.89. In absolute values, the errors were 51.1/43.5, 5.7/4.8, and 12.3/9.2 N for the RMS error/MAE of the vertical, mediolateral, and anteroposterior GRFs, respectively (the bar graph is provided in the Supplementary Materials). The results over the time series (Figure 6(c,d)) show an increase in error compared to the segmented data (the absolute values are shown in the Supplementary Materials).

Looking at the results over the time series (Fig. 6(c,d)), a noticeable increase in error can be observed compared to the segmented. This discrepancy is to be expected as the averaging inherent to segmentation should decrease the influence of outliers. These outliers lead to larger RMS errors whereas the MAE stays similar with the RMS error and MAE are around 10 and 7.1%. By averaging, the influence of outliers (that is larger mistakes in estimation) will be reduced. Such outliers would increase the RMS error more while the MAE is less affected. The 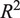 -value tells a similar story as the RMS error and still indicates reasonable explaining power especially, for the vertical and mediolateral GRFs (both exceed 0.8) while the anteroposterior has an 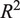 of 0.7. The increase in absolute values also shows a notable decrease in performance with the RMS error/MAE now being 103.8/68.9, 10.1/7.4, and 19.1/14.0 N for the vertical, mediolateral, and anteroposterior GRFs, respectively.

These results show that the HW model outperforms the linear models by a wide margin. Due to these results, the results of the multiday trial will focus on the HW models only.

In addition, the same pair of insoles was reused by the participants even though participants had different shoe sizes. However, there is no clear relationship between the shoe size and estimation performance. There are some outliers in terms of performance with smaller shoe sizes but no clear relationship can be observed, as seen by the low standard error. Implying that the usage of a single size of insole was not detrimental to estimation performance. However, the reason why it did not affect the estimation performance is not known. One reason could be that the sensors were large enough such that the sensor was still compressed even though they were not at the optimal location.

Lastly, the identified models were evaluated in terms of model order. The average number of poles and zeros are shown in Table 1. Because the amount of poles and zeros were set the same for all inputs, only a single value was given. Furthermore, the amount was not rounded to preserve information. It can be observed that the order of both the linear and HW models is similar for the vertical GRF. However, for the mediolateral and anteroposterior GRF, the HW model is of higher order but with less variance. Due to our limited pole-zero combinations for model identification, it is uncertain whether these pole-zero combinations are a general result. However, the poles and zeros are below the maximum indicating that the limited parameter space was sufficient in most cases. The high variance also indicates different participants required different pole-zero combinations. Furthermore, both the optimal order of the segmented and time series have similar means and variances but no clear relationship can be observed.

**Table 1.** Table with the average amount of poles and zeros (with standard deviation) for the Hammerstein-Wiener (HW) and linear (L) models sorted per ground reaction force (GRF). The models were selected separately for the segmented (Seg) and time-series (TS) datasets.

GRF	**L – Seg** [ 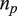 , 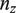 ]	**L – TS** [ 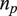 , 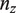 ]	**HW – Seg** [ 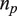 , 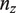 ]	**HW – TS** [ 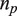 , 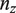 ]
**Vertical**	[4.2 ± 2.4, 2.3 ± 1.2]	[4.7 ± 2.0, 2.8 ± 1.6]	[4.2 ± 1.7, 3.7 ± 1.6]	[4.7 ± 1.3, 3.3 ± 1.2]
**Mediolateral**	[3.1 ± 1.4, 2.3 ± 1.5]	[3.7 ± 1.9, 2.7 ± 1.4]	[5.2 ± 1.3, 2.9 ± 1.1]	[4.9 ± 1.8, 2.2 ± 0.7]
**Anteroposterior**	[3.6 ± 1.6, 2.9 ± 1.8]	[3.3 ± 2.1, 2.3 ± 1.5]	[5.5 ± 1.9, 2.7 ± 1.3]	[6.1 ± 2.0, 2.2 ± 0.8]

### 3.4. Multiday trials

**Figure 7.**
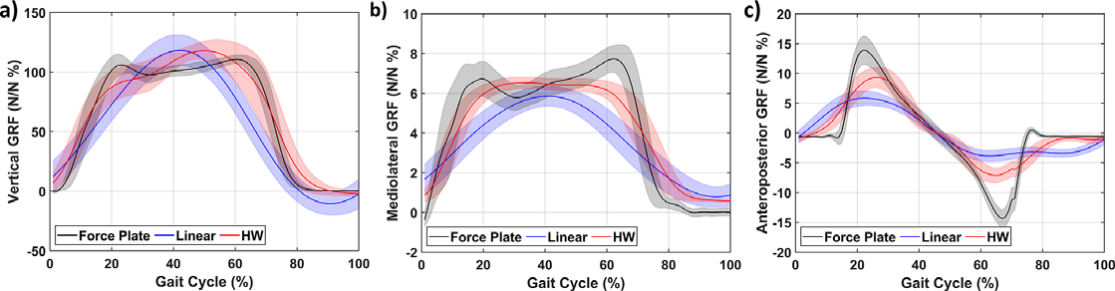
Estimated and measured 3D ground reaction forces for the same participant as Figure 5 for the (a) vertical, (b) mediolateral, and (c) anteroposterior forces a week after model identification averaged based on approximately 115 gait cycles.

The results of the single-day trial are promising. However, having to reidentify models between tests is undesirable. Therefore, the models were evaluated to see how the estimation performance behaves over time. The insoles were calibrated/identified on day 0 (that is from the previous subsection). Subsequently, the participant wore the insoles again, both one day and one week later. The segmented gait cycle results of the GRF for a single participant are shown in Figure 7. It can be observed that the models still approximate the GRF curves to a reasonable degree. However, the anteroposterior GRF does seem to have a less similar shape. Whereas the vertical and mediolateral GRF quality are less affected. Similar to the day of model identification, the linear models are significantly worse than the HW model.

**Figure 8.**
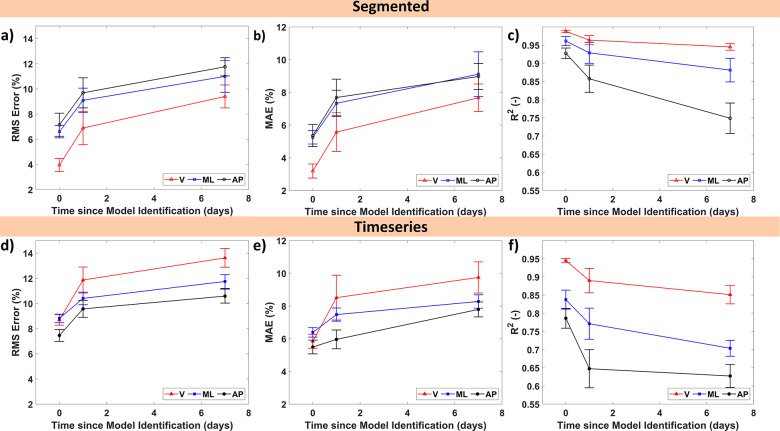
Multiday estimation performance with standard error of the 3D ground reaction forces with V, ML, and AP referring to the vertical, mediolateral, and anteroposterior GRF, respectively. The graphs are separated into the segmented gait cycle (a–c) and time-series (d–f) datasets. The (a,d) RMS error and (b,e) MAE are both shown as a percentage of the force amplitude while (c,f) show the coefficient of determination 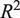 . The day of model identification is considered day 0 with the data on that day only including the four participants that are part of the multiday trial.

The same model metrics were evaluated for both the segmented and time-series data (Figure 8) for the HW models. It can be observed that estimation performance decreases significantly between day 0 and the day after for the segmented data (Figures 8 (a–f)). In contrast, the performance on the day after and a week after seems similar for all metrics. Although the performance a week later is lower, the HW models still have MAEs on average of approximately 8.6% (of the force amplitude) and 10.7% for the RMS error. Similarly, the 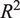 value decreased to 0.95, 0.88, and 0.75 for the vertical, mediolateral, and anteroposterior GRFs, respectively, thereby showing that the estimation performance has reduced but is still decent. While the increase in RMS error/MAE (a figure of it is provided in the Supplementary Materials) reached up to 63.6/24.5, 6.4/5.2, 17.93/13.0 N for the vertical, mediolateral, and anteroposterior GRFs, respectively, which still indicate decent estimation performance.

The time-series data (Figure 8 (c,d)) gives a similar picture with the performance decreasing. A week later, the HW models had a performance of 13.6, 11.8, and 10.6% RMS error for the vertical, mediolateral, and anteroposterior GRFs, respectively. While the MAE was on average 8.6% and the 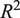 -value also decreased to an average of 0.73 with the anteroposterior performing the poorest (0.63). The increase in RMS error and MAE (see Supplementary Materials) reached up to 121.1 and 81.6 for the vertical GRFs, 11.3 and 8.3 for the mediolateral GRFs, and 23.3 and 16.9 for the anteroposterior GRFs, respectively.

Thereby indicating much poorer estimation performance but similar to the segmented gait cycle, the performance drop between the day and the week after is significantly less.

For both, the segmented and time-series data the MAE still indicates decent estimation performance (9.7% at the most). Similarly, the 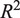 values still show a decent value while the RMS is still below 15% for all GRFs.

## 4. Discussion

This work aimed to investigate the feasibility of foam-like 3D-printed sensors for estimating 3D GRFs. The overall results presented in the previous section indicate the sensors can provide a reasonable estimate of the GRFs when fed into identified HW models. This section will discuss the results in the context of GRF estimation and other insoles in the literature.

### 4.1. Ground reaction force estimation

Both the sensor behavior and GRF estimation performance (Figures 4 to 8) show promise. The raw sensor data shows a correlation between the sensor behavior and the gait cycle. While the need for a model-based solution is corroborated by the hysteresis seen in the cyclic experiment (Figure 3). This result implies that the raw sensor data can be used to estimate the gait cycle. Furthermore, the correlation with the gait cycle could indicate that the GRFs (also related to the gait cycle) can be estimated.

Similarly, the averaged GRFs (Figures 5 and 7) correlate well with the vertical GRF. This correlation can be exploited to estimate the gait phase using the estimated models. Besides estimating the gait phase from the vertical GRFs, the estimation performance of the GRFs seems reasonable (RMS error on average below 10%). The separation in time series and segmented does seem warranted as the averaged data perform much better, indicating that the averaging indeed seems to obfuscate outliers, which can be detrimental to closed-loop control. This result implies that when evaluating the estimation quality of the insoles, one does need to take into account whether the time series or segmented suffices for the intended application. Furthermore, the results imply that the HW model is significantly better at providing force estimates than linear models.

In general, the degradation over time is notable. However, the results imply that the estimation performance degrades the most from 0 to 1 day after model identification. Evaluation over more days would be required to investigate whether the HW model’s estimation performance stabilizes or further degrades. Furthermore, the segmented gait cycle and time series both show similar qualitative degradation. However, the segmented gait cycle does seem to degrade more significantly as ratiometrically its increase seems larger. Comparing this degradation over time with insoles that use machine learning with commercial FSRs is difficult, as the prediction performance over time was not reported in [25, 26, 27].

Solving this degradation over time would make the proposed sensors more feasible. The reason why the performance degrades is not fully understood. A possible reason is that the piezoresistive sensors drift. This drift is also seen in commercial FSRs, which is hypothesized to be due to the carbon particles and polymer matrix acting as a resistor–capacitor circuit [33]. This capacitive drift can charge the sensor over the course of a day changing the sensor behavior. For instance, by changing the value when no-load is applied [29]. Such behavior could explain why the change between the day and the week after is less significant as the majority of walking trials were in the first week. Leading to a more significant charge on days 0 and 1 since model identification but less a week later. Such drift could be remedied by, for instance, a drift compensation circuit such as proposed in [33].

We observed a charging-like behavior in the cyclic experiment (Figure 3(a)). It is unsure whether the models captured this behavior properly as we removed the drift. This function would have removed the charging behavior, as on smaller time scales it is approximately a linear trend. Investigating how to incorporate this behavior in the model would be essential for consistent estimation performance over longer time scales from beginning to end.

Although the cyclic experiment reached forces of more than 500 N, these forces are still less than those encountered during walking. In addition, during the experiments, only the foam-like sensor was compressed, while the insole provided additional stiffness during walking. In future work, a stiffer structure added around the sensor could increase sensitivity at the force range of interest. In addition, such a mechanical modification would also increase the range. In addition, the strain rate plays a role in the sensor’s behavior as it behaves viscoelastically. The 800 mm/min used is much faster than during actual walking, which could have impacted our results. A more thorough investigation would be required to see how the sensor is affected by the load cases and is related to its sensitivity and range.

Another complicating factor regarding estimating the sensitivity and range is that the sensor is fed into a model and combined with other sensor outputs. It is unclear how an individual sensor’s sensitivity and range relate to the overall sensitivity and range of the model. Especially, as the range of a single sensor could be insufficient, yet the GRF estimation was still decent. However, as seen in Figures 5 and 7, the vertical GRF’s peaks are not captured well, which could be due to being at the limits of the range of the heel sensor.

Furthermore, a limitation of the current study is that the number of cycles was limited. Although our insoles survived >6,000–16,000 cycles (cyclic test+walking trials), this number of steps is still limited compared to the daily number of steps. Nevertheless, it is encouraging for 3D-printed sensors. In a future study, further investigation into the long-term behavior is required to conserve the RMS error and MAE. One approach would be model-based compensation by understanding the behavior of the 3D-printed sensors over time.

Two limitations in this study are the use of personalized models and the evaluation of estimation performance over a week for a walking task. The former means that models needed to be identified nine times. Although personalized models could be useful for patient-specific insoles, this approach might not benefit patients for whom calibration is too taxing or walking is difficult due to an injury. Such patients would benefit from a generalized ready-to-use model instead of a personalized one. Investigation into generalized models is an important next step for such patients. In addition, the focus on only a walking task limits the applicability of results. In the future work, more complex tasks such as walking up stairs and jumping could be added to see how the HW models generalize.

In addition, we limited the structure of the identified model. Although the identified HW models provided good estimates, the structure is likely not optimal. Specifically, the choice to keep the same amount of poles and zeros for each input could be suboptimal. Further investigation could be performed by identifying a state space implementation of the HW model, which could lead to models with a more optimal combination of poles and zeros. This change could lead to lower model complexity and better GRF estimates.

Lastly, a limitation in our study was that we only included able-bodied participants. Although our results show promise, a question remains how a pathological/abnormal gait would affect the GRF estimation performance. Especially as we used fewer sensors than other FSR-based insoles, which used six or more sensors [25, 26, 27]To investigate the effect on estimation performance, a wider population could be included such as the elderly, individuals with a gait impairment or children.

### 4.2. Insole comparison

The developed insoles are capable of estimating the 3D GRFs using the personalized identified models. To put the acquired model metrics into context, the insole will be compared to several other force-sensing insoles. The focus will primarily be on FSRs as these are also based on piezoresistive sensing.

Firstly, in terms of performance, the presented insoles can estimate the gait phase by using the estimated GRFs, thereby making our insoles on par with insoles such as [12, 22, 34] that can provide gait cycle data. However, our insoles can also estimate GRFs. A table comparing our results with four other insoles is shown in Table 2. Only the scaled RMS is used, as this makes comparison possible regardless of subject. Furthermore, the data of the segmented gait cycle is used as this is often used in other works as well whereas none of the works compare over multiple days so that comparison was not possible.

**Table 2.** Table with selected works of sensorized insoles. Estimation evaluation refers to the amount of days that were evaluated. The cyclic testing refers to any test of the sensors under a cyclic force. Both the RMS and 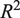 display (in order) the vertical, mediolateral, and anteroposterior GRF results. Treadmill refers to an instrumented treadmill with embedded force plates. Notes:^1^: knee angle sensor,^2^: piezoelectric film sensors

Metric	Our insole	**FSR** [25]	**FSR and angle sensor** [26]	**Pneumatic** [15]	**FSR and piezoelectric** [27]
Sensor	3D printed	Commercial	Commercial	Custom	Commercial
number of sensors	4	6	6 + 1^1^	4	4 + 4^2^
principle	Piezoresistive	Piezoresistive	Piezoresistive and knee angle sensor	Pressure	Piezo-resistive and electric
Model	HW	Artificial neural network	Random Forest	Hysteresis compensator	Support vector regression
No. Participants (−)	9	8	17	1	11
Estimation evaluation (Days)	7	Not reported	Not reported	1	Not reported
Cyclic testing (Cycles)	10,000	None	None	1,000	1
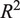 (−)	0.94, 0.88, 0.85	0.96, n/a, n/a	0.91, 0.59, 0.91	0.98*, n/a, n/a	not reported
RMS error (%)	6, 10, 9	5, n/a, n/a	8, 14, 7	32, n/a, n/a	8, 9, 9
RMS error (N)	51,5.7,12	34	66, 11, 23	186,n/a,n/a	Not reported
Average weight (kg)	80.1	73	66.6	54	66.2
data acquisition	Treadmill	Treadmill + Force plate	in-ground Force plate	Treadmill	Treadmill

Comparatively, our sensors are printable whereas the other insoles relied on the assembly of commercial FSRs. This structural integration should make it less noticeable to the user. Furthermore, the ability to 3D print the sensor allows for customization that can be beneficial for patient-specific applications such as those with musculoskeletal ailments [18] or diabetic patients [31]. The ability to print a foam-like structure that can be mechanically programmed [30] to provide mechanical support where needed while also acting as a sensor can be useful for patient-specific insoles with integrated sensing. Furthermore, the printing of an insole would require around 32 grams of material. Assuming a cost of 5–100 euro/kg for elastomeric pellets, the total price would range from 0.37 to 6.20 euros for both insoles. This cost although preliminary indicates that material wise it can be an affordable yet patient-specific solution.

Moreover, being lightweight and comfortable are important for user acceptance. Our insoles, made from a soft foam-like material, ensure comfort, while their weight of just 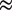 31.1 grams (excluding electronics) makes them lightweight and suitable for extended use. This value is difficult to put into context, as other researchers often do not report the weight of the insole. An exception is [27] they only report the weight of the total package, not the insoles.

All insoles used different models for estimation. This difference makes absolute comparisons of the metrics difficult as the comparison is between the combination of a modeling/learning/identification approach and sensor not merely the sensor. In addition, comparing degradation over time with insoles that used machine learning with commercial FSRs was not possible, as none of the other works reports the estimation performance over time.

It can be observed that our insoles perform comparably in terms of the coefficient of determination 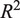 for the vertical GRF. The insole of [15] comes near our 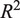 but was only based on their repeatability testing, not GRFs. Furthermore, their evaluation was based on the time series data, which is still worse than ours. The scaled RMS error values are in the same order of magnitude for all the piezoresistive sensors.

The mediolateral and anteroposterior GRFs were also estimated in [26]. Their 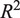 is lower than ours just like their RMS error for the mediolateral GRF. Whereas their anteroposterior force estimation [26] is better compared to ours. In contrast, [27] is similar to ours for all RMS errors but did not report 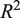 .

A similar pattern is observed with the RMS error in absolute values as shown in [25, 26, 27] while the results in this study outperform those reported in [15]. However, interpreting these results is challenging as all studies have differences in the number of participants and their body weights making comparisons between absolute values difficult to generalize. Nevertheless, the 3D-printed insoles performed comparable to the others.

Overall, our printed sensors estimate the GRFs comparable to other insoles. Even though our insole uses two sensors less than the other two FSR-based insoles. Due to the comparable performance, our insole combined with system identification seems a promising candidate for further investigation. One aspect that is comparatively missing is estimating the center of pressure (done in [25, 26]) and pressure distribution. Both can be a topic for future research.

In addition, the data acquisition method is an important point of discussion. All the insoles in Table 2 use a set of flexible or soft sensors that compress and bend. Their raw data is then fed into a model that converts it to an estimate of the GRFs. The advantage of this approach is that the sensor design is relatively straightforward. Unfortunately, it also means that a ground truth is required to train a model.

This ground truth requires complex systems such as force plates. This requirement makes it more difficult to apply these insoles in practice. One possible solution is to use multiaxial sensors such that the models can be acquired externally, which increases sensor complexity but could simplify calibration. However, the distributed nature of the forces over the foot could make this nontrivial. For instance, in [27], they used both FSRs and piezoelectric film sensors combined with support vector regression for GRF estimation and saw an improvement. Another approach used by [26] is to complement the model with user data such as shoe size and body weight for model generalization. However, it was not reported how the model generalizes to new insoles or the training would need to be performed again. Such approaches could also be investigated in the context of 3D-printed (foam-like) sensors to benefit from personalization and improved estimation as part of future work. Our results suggest that personalized models may outperform generalized models.

In Table 3, an overview of different insole technologies is provided and evaluated on different aspects. The foam-like sensors have the advantage of customization and scalability as other 3D-printed insoles while also being soft. Although the F-Scan has a better spatial resolution, the scalability of 3D-printed sensorized insoles could attain equivalent or higher resolution by adding more sensors.

**Table 3.** Table comparing selected insole approaches from literature with GRF dimension based on the highest in the references. Abbreviations: 3DP:3D-printed, PR: piezoresistive, PC: piezocapacitive, flex:flexible, EOF: Embedded optical fiber, KF+: Kalman Filter and dedicated algorithms, ML: machine learning, Prop: proprietary. Notes *: more electrodes can be added during printing, ^1^: capacitive measurements require specific electronics and shielding, ^2^: plantar pressure out of the box but with deep learning 3D possible [35].

Technology	Softness	Customizable	GRF estimated	Cost	Data conversion	Spatial resolution	Electronics
Foam-like (ours)	Soft	Yes	3D	Low	HW	Scalable*	Simple
DP – PR [24, 22]	Flex to Soft	Yes	Raw data-only	Low	n/a	Scalable*	Simple
DP – PC [20, 23]	Flex	Yes	Raw data-only	Low	n/a	Scalable*	Complex^1^
DP – EOF [21]	Flex to Rigid	Yes	Raw data-only	Mid	n/a	?	Complex
Pneumatic [15]	Flex	?	V	Low	Custom	?	Simple
IMU [8]	n/a	n/a	3D	Mid	KF+	None	Simple
FSR [25, 26, 27]	Flex	Limited	3D	Mid	Mid	Scalable*	Simple
DoF Loadcell [10]	Rigid	No	3D	Mid	Custom	None	Simple
F Scan Go (TekScan, USA) [35]	Flex	Some	1/3D^2^	High	Prop/ ML^2^	High	Prop

The current number of sensors is low compared to state-of-the-art products such as the F-Scan (TekScan, Inc., USA), which makes it unlikely to capture pathological gaits. Future work would be to sensorize the entire insole by making it a single large 3D-printed (porous) sensor with a smart electrode pattern spread over its surface. A challenge in such an insole includes where to place the electrodes and get useful data over the entire area. Another approach would be to fabricate multiple smaller sensors. Both of these approaches are feasible within the presented framework as the InFoam method can scale up the area/number of electrodes/sensors while more inputs can be fed into HW models. Realizing an insole with more sensors would increase the spatial resolution and could make it possible to capture pathological gait. In addition, this sensorizing can aid in estimating the center of pressure, which was not investigated.

However, an important design question is where to place the sensors. Previous works showed comparable results to our insole with more FSR sensors (four to six) combined with other sensors [25, 26, 27] while the F-Scan has even more sensors. However, it is unclear whether adding more sensors is optimal or whether specific points on the foot are more important/informative for the GRFs and centers of pressure. Optimizing these locations could preserve estimation performance while minimizing model complexity and the number of sensors. Such an approach could benefit from the ability to 3D print the insoles based on a 3D scan of the patient’s foot for a patient-specific insole with optimal sensor locations. By 3D printing a desired stiffness pattern, this sensorized insole could also aid in preventing ulcers [31].

## 5. Conclusion

Sensorized insoles are a useful tool to gain insight into aspects related to gait and health monitoring. For such insoles to be used in daily life, they need to be, among others, comfortable and lightweight. Within this work, the feasibility of 3D-printed foam-like sensors for sensorizing insoles was investigated. The soft and foam-like nature of these sensors makes them comfortable and lightweight.

Our findings indicate that the 3D-printed foam-like sensors combined with identified personal HW models can estimate all three GRFs with reasonable accuracy (RMS 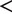 10% on the day itself) and decent accuracy a week later (on average 11% and 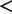 13%), with performance comparable to FSRs combined with machine learning. This similarity in estimation performance supports the feasibility of this approach. Furthermore, the estimation consistency over multiple subjects indicates that the combination of 3D printing and system identification is an interesting opportunity for wearable sensors.

A limitation of the presented approach is the need for an instrumented treadmill for calibration. Two approaches could be investigated for simplifying usage. An option is to use direction-specific sensors that can sense the vertical, mediolateral, or anteroposterior forces, which could perhaps be calibrated using a tensile tester or force plate instead of a treadmill.

The current insoles showed a decrease in estimation performance over multiple days. The next step would be to investigate the compensation strategies, such as modeling the sensor’s behavior over a longer time or re-calibrating after x days (with x to be determined through a longer study).

Lastly, real-world scenarios such as running or walking up stairs could be investigated to evaluate the GRF estimation performance in more realistic scenarios. An open question herein is whether the GRF performance generalizes well to other scenarios or whether the models need to be specialized.

## Supporting information

Willemstein et al. supplementary material Willemstein et al. supplementary material

## Data Availability

The datasets generated during and/or analyzed during the current study are available from the corresponding author upon reasonable request.

## Supplementary material

The supplementary material for this article can be found at http://doi.org/10.1017/wtc.2024.23.
