# Supplementary material for: A feasibility study on using soft insoles for estimating 3D ground reaction forces with incorporated 3D-printed foam-like sensors
Source: Wearable Technol. 2025 Jan 23;6:e3. doi: 10.1017/wtc.2024.23 (PMC11810523; doi:10.1017/wtc.2024.23)
Supplement: Willemstein et al. supplementary material [file S2631717624000239sup001.docx]

**Supplementary Materials**

**A Feasibility Study on using Soft Insoles for estimating 3D Ground Reaction Forces with incorporated 3D Printed Foam-like Sensors**

Nick Willemstein Saivimal Sridar Herman van der Kooij Ali Sadeghi*

**Overview:**

1. Sensitivity Plot
2. Force Value RMS error and MAE
3. Behavior over Time with Force

## **1. Sensitivity Plot**

The sensitivity plot is shown below in Figure S1. It can be observed that the sensitivity reduces with cycles 1000 and 10000 being more similar. Furthermore, the range seems to increase over the cycles through this reduced sensitivity.

**
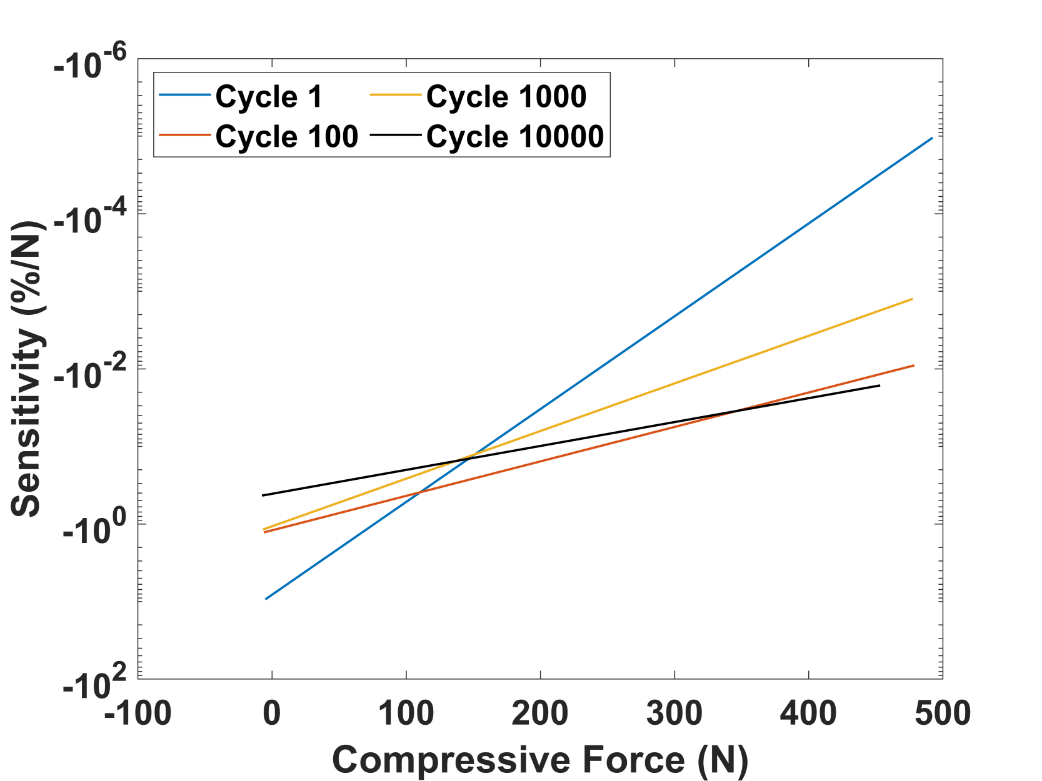
**

Figure S1: The sensitivity over the force for multiple cycles in units of resistance change.

## **2. Force Values of** **RMS error and MAE**


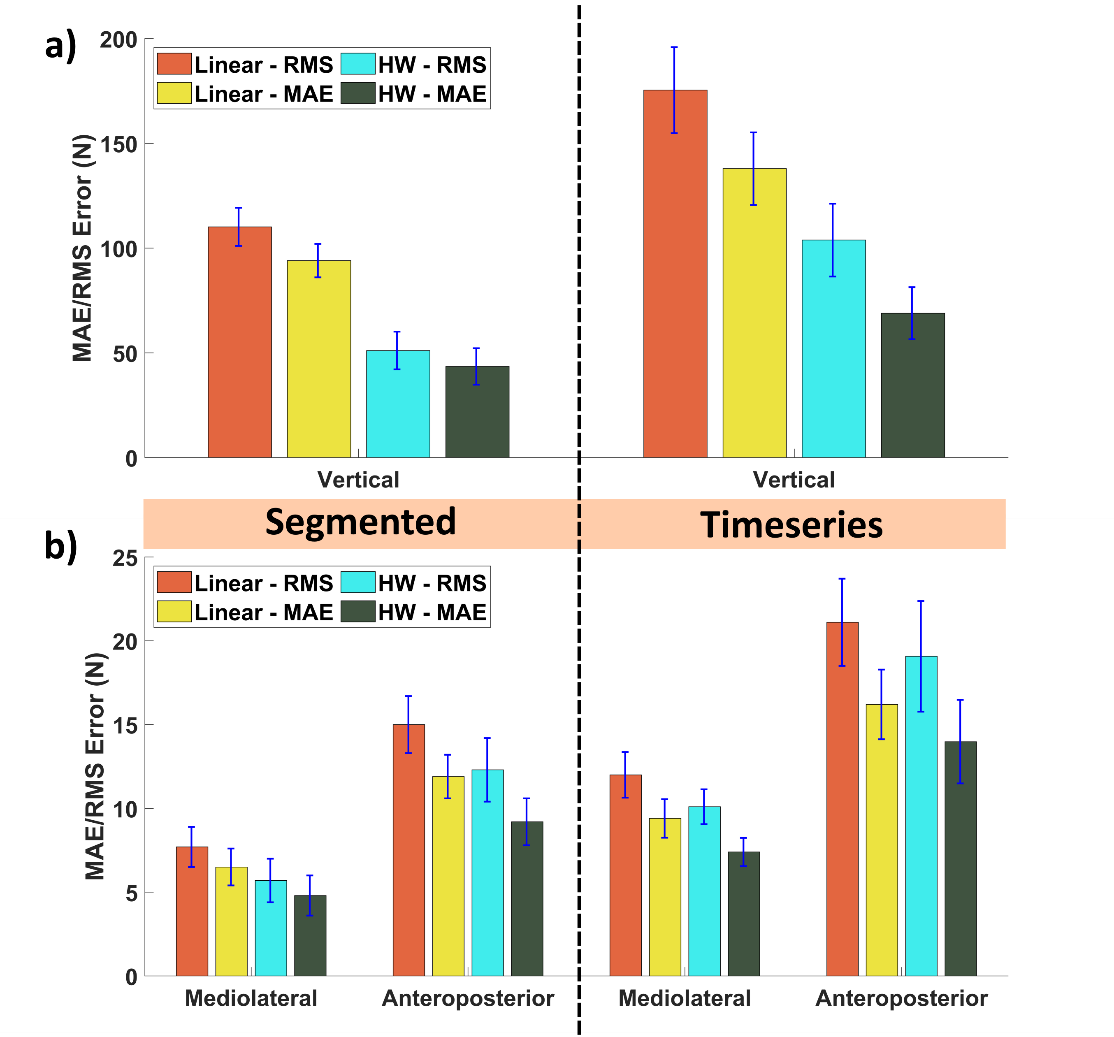


Figure S2: a,b) The absolute errors of the models for the (a) vertical and (b) mediolateral and anteroposterior GRFs

## **3. Performance Degradation in Absolute Values**


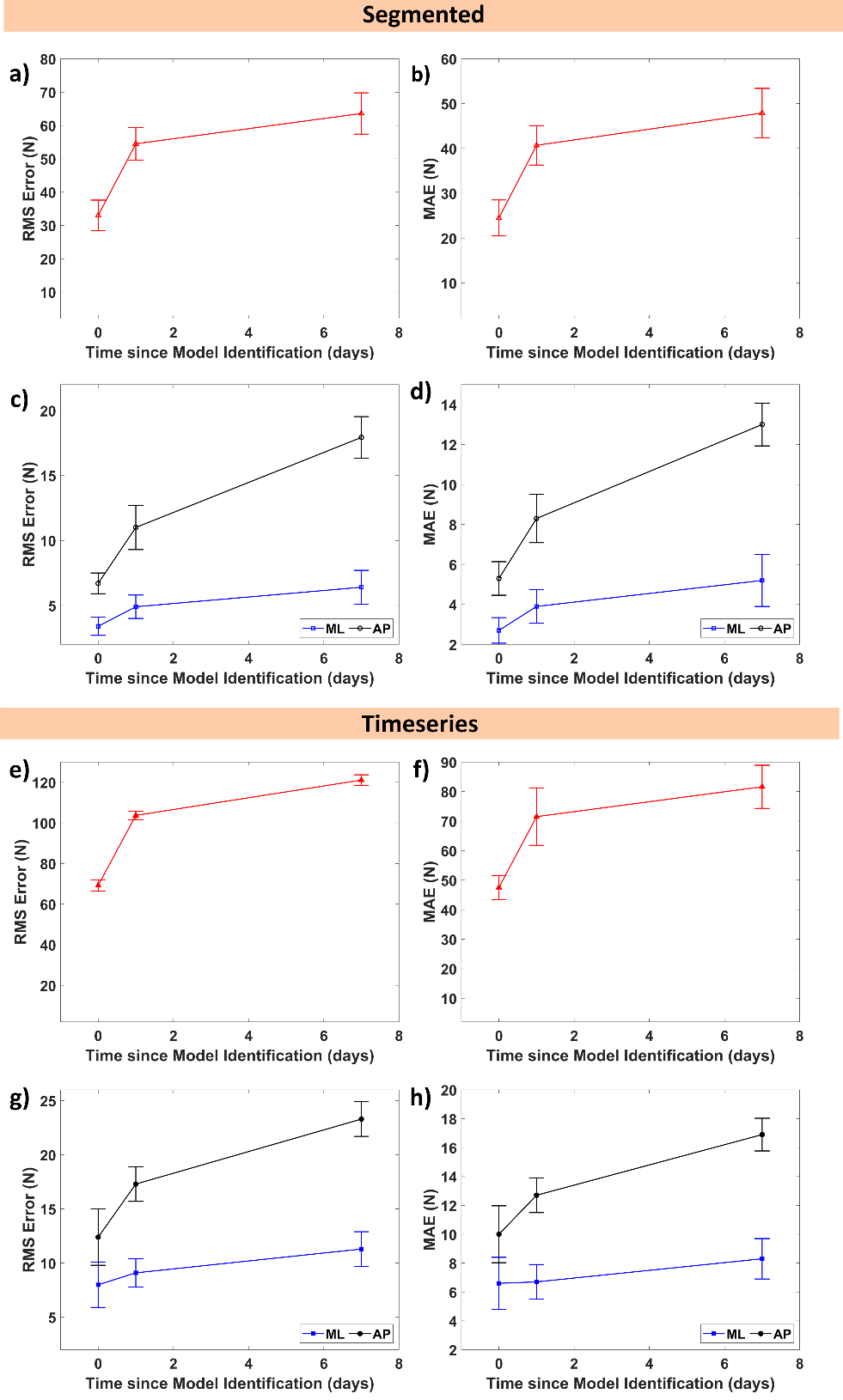


Figure S3: The estimation performance decay over time for (a-d) segmented and (e-h) timeseries data.
